# The Effect of Essential Oils from Asteraceae Plants on Behavior and Selected Physiological Parameters of the Bird Cherry-Oat Aphid

**DOI:** 10.3390/molecules29071673

**Published:** 2024-04-08

**Authors:** Paweł Czerniewicz, Hubert Sytykiewicz, Grzegorz Chrzanowski

**Affiliations:** 1 Institute of Biological Sciences, Faculty of Natural Sciences, University of Siedlce, Prusa 14, 08-110 Siedlce, Poland; hubert.sytykiewicz@uws.edu.pl; 2 Institute of Biotechnology, University of Rzeszow, Zelwerowicza 8B, 35-601 Rzeszow, Poland; gchrzanowski@ur.edu.pl

**Keywords:** essential oils, *Rhopalosiphum padi*, anti-settling activity, probing behavior, digestive enzymes

## Abstract

Essential oils (EOs), including those from the Asteraceae plants, have been shown to have promising insecticidal activity against a wide range of insect pests. Understanding the mechanism of action of EOs is one of the studied aspects. The present study aimed to evaluate the effect of essential oils from *Achillea millefolium*, *Santolina chamaecyparissus*, *Tagetes patula* and *Tanacetum vulgare* on the settling and probing behavior of the bird cherry-oat aphid (*Rhopalosiphum padi* L.). In addition, the effect of the oils on the activity of such enzymes as trypsin, pepsin and *α*- and *β*-glucosidase involved in the metabolism of proteins and sugars of the insects was examined. The leaf-choice bioassays demonstrated that the studied EOs limited aphid settling for at least 24 h after the treatment. The application of EOs also inferred with aphid probing behavior by reducing the total probing time and total duration of phloem sap ingestion. Aphids spent more time in the search phase due to an increase in the number and total duration of pathway phases. Moreover, the activity of the studied proteases and glucosidases significantly decreased in *R. padi* females exposed to the EOs. The enzyme inhibition varied depending on the applied oil and exposure time. Generally, the EOs with stronger deterrent activity also showed higher inhibitory effects. The results suggest that the tested EOs disrupt key digestive processes in *R. padi* which may be an important factor determining their aphicidal activity.

## 1. Introduction

Plant secondary metabolites play a key role in plant-insect interactions and very often function as a chemical defense against herbivorous insects. The defensive properties of these compounds have been exploited for the development of products to control different groups of pests. Among the plant products, essential oils (EOs) and their constituents seem to be one of the most promising [1,2]. Essential oils are composed of a complex mixture of volatile organic compounds and are obtained mainly by steam distillation of various parts of aromatic plants, such as flowers, leaves, roots and stems [3]. As natural substances, they show a wide range of biological activities against insects, including toxic, repellent, and deterrent effects, as well as inhibition of oviposition and reduction in growth and reproduction [4,5,6,7]. Therefore, essential oils are often called natural pesticides. Compared to chemical insecticides, the use of EOs in pest management has many advantages. They are often more selective, easily biodegradable and have less impact on non-target organisms. Moreover, due to their multi-component nature, the probability of developing a resistant population is very low [8].

Aphids are worldwide distributed pests that cause serious losses to many agricultural and horticultural crops. During feeding, they damage plants directly by sucking phloem sap and indirectly through the excretion of honeydew, which covers the leaves and enables sooty mold development. Indirect damage is also caused to a large extent by the transmission of plant pathogenic viruses [9]. The bird cherry-oat aphid (*Rhopalosiphum padi* L.) is a global pest of cereals and is listed among the fifteen aphid species of the most agricultural importance worldwide. Due to their high reproductive capacity, they can reach high population levels in a relatively short period of time. Apart from direct damage and yield loss, *R. padi* can also efficiently transmit destructive plant viruses, especially Barley yellow dwarf virus (BYDV), for which it is the most important vector [10,11]. Elimination or at least reduction of aphid feeding on plants may reduce the virus infections and yield losses.

The available literature indicates that the use of EOs and their constituents can significantly contribute to the reduction in the occurrence of pest populations in agricultural crops. Among aromatic plants, those from the Asteraceae family have received considerable attention [5]. Essential oils and extracts from these plants exhibit a broad spectrum of activity against various groups of insects, including aphids. Almeida et al. [12] demonstrated that essential oil from yarrow (*Achillea millefolium*) had a repellent effect on *Hoplocampa testudinea* adults and reduced its oviposition on the host plants. This EO was also highly toxic to *Aphis gossypii*, while sublethal concentrations reduced the insect’s fecundity [13]. Essential oil obtained from tansy (*Tanacetum vulgare*) had insecticidal properties against *Acrobasis advenella* (Zinck.) and significantly worsened the biological parameters of this pest [14]. Moreover, tansy extract had deterrent and insecticidal activities against *Aphis fabae* and *Sitona lineatus* [15]. Also, the strong antifeedant activity of *Santolina chamaecyparissus* essential oil against *R. padi* has been demonstrated [16].

Despite current interest in the insecticidal properties of essential oils, their mode of action has not been completely elucidated. It should be emphasized that a comprehensive understanding of the mechanisms by which these active metabolites affect insect behavior and physiology is necessary for their effective and safe use in pest control. Essential oil components exert their activity on insects primarily through neurotoxic effects involving the inhibition of acetylcholinesterase (AChE) and an effect on the octopamine synapses and GABA receptors [17,18]. Our previous research has shown that several EOs from Asteraceae plants have toxic properties against aphids *R. padi* and *M. persicae*. The oils reduced the aphids’ biological parameters and significantly inhibited the activity of AChE and Na^+^/K^+^-ATPase, the key enzymes of the insect nervous system [19,20]. However, an important aspect of the insecticidal activity of EOs is also their effect on aphid settling and feeding behavior. The interferences with aphid feeding and host plant selection strategy may result in a decline in food consumption. This limitation may consequently cause the rejection of a plant, may negatively affect the development of the insect, and finally may even lead to its death [21]. Moreover, biochemical studies showed that active constituents of EOs affect the digestive performance in insects through changes in enzyme activities. Any disruption in the activity of digestive enzymes limits the availability of nutrients for biological requirements [14,22]. Proteases are considered one of the most important enzymes in insects, as they hydrolyze the peptide bonds in dietary proteins to liberate the amino acids needed for growth, survival, and reproduction [23]. Glucosidases, such as α- and β-glucosidase, are a type of digestive enzymes that have a critical role in the final stages of carbohydrate digestion [24].

To date, most studies have focused on the direct toxic and/or repellent effects of essential oils on insects, while there are very limited data on uncovering the complex mechanisms underlying their toxicity. Therefore, the undertaken analyses were focused on the assessment of the effects of selected essential oils from the Asteraceae family on changes in the behavior of *R. padi* during the different feeding phases and the influence on the activity of digestive enzymes which are an important determinant of proper food assimilation. The aim of the present research was to determine the effect of EOs obtained from *A. millefolium*, *S. chamaecyparissus*, *Tagetes patula* and *T. vulgare* on the settling and feeding behavior of the bird cherry-oat aphid. The behavioral background of the anti-settling and feeding deterrent activity was investigated using a classical choice test and the electrical penetration graph (EPG) technique, respectively. Another objective of this study was to verify the thesis that the tested oils disturb the functioning of the digestive enzymatic systems in aphids by altering the activity of trypsin, pepsin and α- and β-glucosidase.

## 2. Results

### 2.1. Settling Inhibition Activity

The leaf-choice bioassays showed that significantly fewer aphid females settled on leaves treated with EOs than on the controls (Table 1). The observed settling inhibition effect was maintained throughout the twenty-four-hour period; however, it decreased with the passage of time. Among the tested oils, the oil obtained from *A. millefolium* exhibited the highest settling inhibition activity, ranging between 68 and 81%. Essential oils from *S. chamaecyparissus* and *T. vulgare* showed moderate activity against *R. padi*, with SI values in the range of 64–76% and 56–66%, respectively. The weakest settling inhibition activity was noted after the application of essential oil from *T. patula*, where SI values ranged from 32 to 57%.

### 2.2. Effect of EOs on Probing Behavior of R. padi

EPG recordings indicated that treatment with the studied Asteraceae EOs clearly affected the probing behavior of the bird cherry-oat aphid on wheat seedlings (Table 2). In comparison to the control plants, the number of probes increased by about 60% and 100% after treatment with essential oil from *A. millefolium* and *T. vulgare*, respectively. The total duration of stylet penetration was significantly shorter (5–11%) after the application of the tested essential oils, except for *T. vulgare* oil. Exposure of *R. padi* to essential oils from *A. millefolium* and *S. chamaecyparissus* prolonged the time from the start of EPG recordings to first stylet penetration by 270% and 150%, respectively. The number of pathway phases significantly increased after treatment with essential oils from *A. millefolium*, *T. vulgare*, and *S. chamaecyparissus;* however, the total duration of the pathway phase was longer than in the control only in the case of *T. vulgare oil*. Moreover, the application of these two oils (*A. millefolium* and *T. vulgare*) led to a reduction in the total duration of the phloem phase (~35% reduction), as well as the phloem phase index was significantly lower. The total duration of phloem salivation increased only after treatment with essential oil from *S. chamaecyparissus* (+88%), whereas the time from the start of probing to first salivation was prolonged after spraying with *T. vulgare* oil (+116%). The studied EOs, apart from *T. patula*, also caused a significant decrease in the total duration of phloem sap ingestion. The largest decrease was observed after treatment with *T. vulgare* oil (38%), followed by *A. millefolium* (34%) and *S. chamaecyparissus* (22%). Additionally, the application of *T. vulgare* oil led to an increase (76%) in the time from the start of EPG to the first sustained phloem ingestion (E2 >10 min). Analysis of the results concerning the parameters related to exploratory cell punctures showed that the total number of potential drops (pd) tended to be higher after treatment with the tested EOs; however, a significant effect was confirmed only for *T. vulgare* oil (34%). For the different pd sub-phases, the only significant difference in relation to control was in the duration of sub-phase II-3, which was prolonged by 41% on plants treated with *T. vulgare* oil.

### 2.3. Effect of Essential Oils on Aphid Enzymes

**Figure 1:**
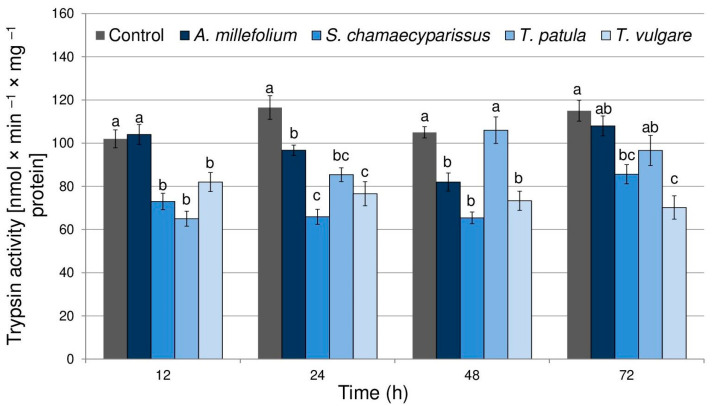
The activity of trypsin (mean ± SE) in apterous females of *Rhopalosiphum padi* after treatment with selected Asteraceae essential oils. Different letters indicate significant differences for each time period separately at *p* < 0.05 (Tukey’s test).

The obtained results showed that treatment with the tested Asteraceae EOs affected the activity of trypsin in *R. padi* tissues (F_(4,40)_ = 47.29, *p* < 0.001) (Figure 1). Moreover, time had a significant effect on the enzyme activity (F_(3,40)_ = 4.77, *p* < 0.01). There was also a significant interaction between treatment and time in trypsin activity (F_(12,40)_ = 6.35, *p* < 0.001). Compared to the control, the enzyme activity significantly decreased after the application of the tested essential oils during almost all experimental periods, except for the oil of *A. millefolium* at 12 and 72 h and for *T. patula* oil at 48 and 72 h. The highest inhibition of trypsin activity within aphid tissues was shown after treatment with essential oil from *S. chamaecyparissus* (26–43%) and *T. vulgare* (19–39%).

**Figure 2:**
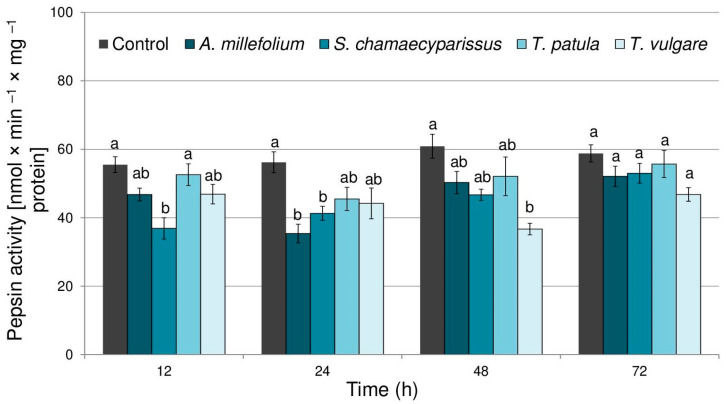
The activity of pepsin (mean ± SE) in apterous females of *Rhopalosiphum padi* after treatment with selected Asteraceae essential oils. Different letters indicate significant differences for each time period separately at *p* < 0.05 (Tukey’s test).

The activity of pepsin in aphids treated with essential oils significantly changed depending on the tested oil (F_(4,40)_ = 14.59, *p* < 0.001), time (F_(3,40)_ = 6.82, *p* < 0.001) and there was a significant interaction between treatment and time (F_(12,40)_ = 2.21, *p* < 0.05). Application of the tested essential oils elicited a decrease in pepsin activity within tissues of the bird cherry-oat aphid (Figure 2); however, significant changes, when compared to the control, were observed only after treatment with essential oil from *S. chamaecyparissus* at 12 and 24 h (27–33% decrease), for essential oil from *A. millefolium* at 24 h (37% decrease), and for *T. vulgare oil* at 48 h (39% decrease).

**Figure 3:**
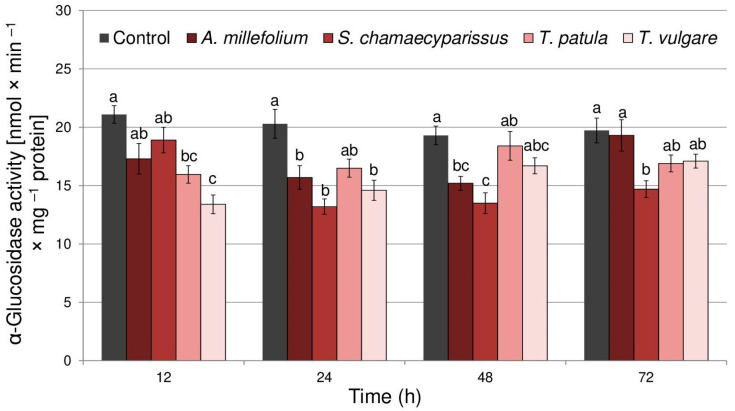
The activity of α-glucosidase (mean ± SE) in apterous females of *Rhopalosiphum padi* after treatment with selected Asteraceae essential oils. Different letters indicate significant differences for each time period separately at *p* < 0.05 (Tukey’s test).

Both the oil treatment (F_(4,40)_ = 19.91, *p* < 0.001) and time (F_(3,40)_ = 3.59, *p* < 0.05) significantly affected the activity of α-glucosidase in *R. padi*. However, there was no significant interaction between the treatment and time (F_(12,40)_ = 1.89, *p* > 0.05). Application of the tested essential oils generally caused decreases in activity of α-glucosidase within tissues of *R. padi*, especially during the first two days after exposure (Figure 3). The highest reduction in enzyme activity was shown after the application of essential oils from *T. vulgare* at 12 h (36% decrease) and *S. chamaecyparissus* at 24 h (35% decrease). Finally, at 72 h after treatment, only essential oil from *S. chamaecyparissus* significantly decreased the activity of α-glucosidase in relation to non-treated insects.

**Figure 4:**
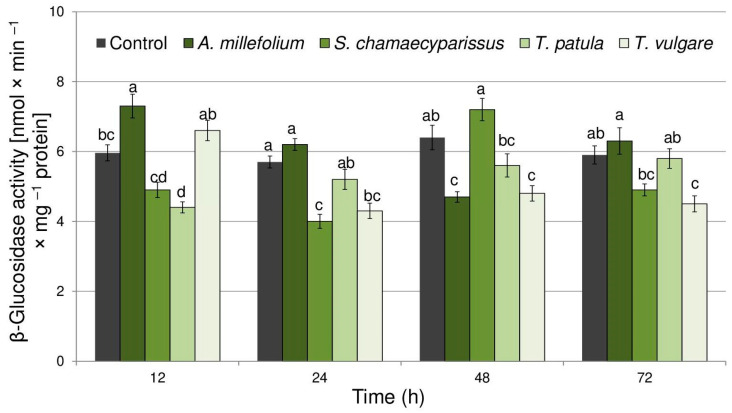
The activity of β-glucosidase (mean ± SE) in apterous females of *Rhopalosiphum padi* after treatment with selected Asteraceae essential oils. Different letters indicate significant differences for each time period separately at *p* < 0.05 (Tukey’s test).

Treatment with the tested essential oils also significantly affected the activity of β-glucosidase within tissues of the bird cherry-oat aphid (Figure 4). The enzyme activity changed with treatment (F_(4,40)_ = 13.91, *p* < 0.001), time (F_(3,40)_ = 8.23, *p* < 0.001) and the interaction treatment × time (F_(12,40)_ = 15.32, *p* < 0.001). Treatment with essential oil from *A. millefolium* resulted in a significant induction (22%) of β-glucosidase activity after 12 h of exposure. At the same time, essential oil from *T. patula* evoked a significant decrease (27%) in enzyme activity. Moreover, significant decreases in the activity of β-glucosidase were shown at 24, 48 and 72 h after treatment with essential oil from *T. vulgare* (24–26%), at 24 h after application of Santolina oil (29%) and at 48 h for *A. millefolium* oil (27%).

## 3. Discussion

Essential oils are complex mixtures of organic compounds including terpenoids, phenols, aldehydes, ketones and esters. These mixtures usually contain two or three main components at relatively high concentrations, whereas other components are present in trace amounts [3]. In our previous research [19], the quantitative and qualitative composition of the tested essential oils were characterized by GC-MS analysis. It was shown that the major compounds identified in essential oil from *S. chamaecyparissus* were artemisia ketone (25.9%), β-phellandrene (18.7%) and vulgarone B (11.6%), while the main components of *T. patula* essential oil were terpinolene (15.8%), limonene (12.5%) and piperitone (9.8%). The essential oil from *T. vulgare* was rich in α-thujone (26.9%), 1,8-cineole (16.8%) and camphor (8.4%), whereas for *A. millefolium*, chamazulene (15.7%), 1,8-cineole (9.5%) and caryophyllene oxide (9.6%) were the most abundant constituents.

The number of aphids that settle and feed on a given substrate is a good indicator of its suitability [25]. Therefore, a conventional settling choice test was carried out to determine the anti-settling activity of the studied Asteraceae essential oils. The obtained results clearly demonstrated that essential oils obtained from *A. millefolium*, *S. chamaecyparissus*, *T. patula* and *T. vulgare* inhibited the bird cherry-oat aphid females from settling on the treated leaves. Previous studies have shown that the settling inhibition of essential oils towards aphids is mainly connected with their repellency, feeding deterrence and locomotion stimulatory activities [26]. Moreover, essential oil components can mask the host plant odors and make it more difficult for insects to locate a suitable host [27]. Such effects increase the time needed to find acceptable food sources, and especially under field conditions, it may lead to significantly longer exposure of the insects to biotic and abiotic environmental factors, increasing their mortality [28]. The studied essential oils showed different levels of anti-settling activity towards *R. padi*. The most active was the essential oil from *A. millefolium* followed by the oils from *S. chamaecyparissus*, *T. vulgare* and *T. patula*. The biological activity of essential oils from these plants has previously been shown against several insect pests including aphids. Essential oil from *A. millefolium* was reported as a strong repellent towards the corn leaf aphid (*Rhopalosiphum maidis* Fitch) [29] and also inhibited settling by the aphid *Myzus persicae* (Sulzer) [19]. Nottingham et al. [27] demonstrated that plant volatiles from tansy (*T. vulgare*) repelled *Aphis fabae* (Scopoli) and *Brevicoryne brassicae* (L.) by masking the attractiveness of the host plant leaves. However, it should be noted that anti-settling activity very often decreases with the passage of time, as was also observed in our bioassays. A similar tendency was also demonstrated by Wróblewska-Kudryk et al. [30] in studies concerning responses of *M. persicae* towards β-thujone and its derivatives. The loss of repellency over time often occurs due to the evaporation of active substances from the leaf surface, since some essential oil components can easily evaporate [31]. Other factors that may contribute to lowering the concentration of active compounds include the degradation of these compounds by plant enzymes and/or their translocation to other parts of the plant [30,32]. One of the promising strategies to overcome these adverse effects is encapsulation, which involves packing the oils in an appropriate material for their controlled release and increased bioavailability [33].

The parameters describing aphid behavior during probing and feeding are good indicators of plant suitability and also interference of probing by different chemical or physical factors in individual plant tissues [21]. Therefore, the EPG technique is often used in research on the mechanisms of antifeedant activity of various xenobiotics, including exogenously applied chemicals like essential oils and their constituents [34,35]. The results of the EPG experiments showed that the treatment of plants with essential oils significantly modified the probing behavior of the bird cherry-oat aphid. Particularly, the essential oils from *A. millefolium* and *S. chamaecyparissus* shortened the duration and increased the latency time of stylet probing. The longer latency time indicates that aphids were reluctant to probe. Such behavior is usually associated with negative factors located at the plant’s surface [34,36], and in our bioassays, it could be explained by the repellent effect of the applied essential oils, which discouraged the aphids from initiating probing. These findings corroborate the relatively high anti-settling activity of yarrow and Santolina oils, which was observed in the choice tests. Furthermore, the results of EPG experiments revealed that on oil-treated plants, especially with *T vulgare* and *A. millefolium*, aphids spend more time in the search phase due to an increase in the number and duration of the pathway phase. Moreover, almost all of the studied essential oils evoked a decrease in the total duration of phloem sap ingestion, and essential oil from *T. vulgare* additionally delayed phloem sustained ingestion, considered the real ingestion phase. Thus, aphids that feed on oil-treated plants ingested the phloem sap for a much shorter period of time than those feeding under control conditions. Similar trends were also reported in the probing behavior of the peach potato aphid after the application of caraway essential oil and its main components—carvone and limonene [37]. On treated plants the probes were short, non-probing intervals were long and the duration of the phloem phase was significantly reduced. Several authors suggest that lipophilic active compounds of essential oils can penetrate the plant surface, pass into deeper tissue layers, and consequently interfere with aphid-feeding behavior [30,34,37]. The disturbances in the feeding process result in a reduction in nutrient intake and lead to a limitation of aphid development [36,38]. The results of our previous study have shown that essential oils from *S. chamaecyparissus* and *T. patula* negatively affected the bionomic parameters of *R. padi* and *M. persicae*. The application of these oils caused the extension of the pre-reproductive period, a reduction in the daily fecundity of aphid females, and a significant decrease in the intrinsic rate of natural increase [20].

Aphids not only weaken plants by sucking out essential nutrients, but they also are vectors of numerous plant viruses. The acquisition and inoculation of distinct types of viruses are strictly associated with various phases of aphid probing and feeding. During brief intracellular probes of epidermal and mesophyll cells, aphids may transmit non-persistent and semi-persistent viruses, whereas the transmission of persistent viruses may occur when aphid stylets reach sieve elements [39,40]. Thus, elimination or at least reduction of penetration of plant tissues by aphids could reduce the virus transmission. Costa et al. [41] argue that reduced feeding time and lower number of individuals that reached the phloem vessels were responsible for reduced transmission of Barley yellow dwarf virus by *Schizaphis graminum*. The results obtained in our study indicated that most of the tested essential oils significantly shortened the time of feeding in phloem, which could potentially limit the rate of spread of phloem-restricted viruses. However, a detailed study on virus transmission needs to be carried out to precisely explore this possibility. It is also worth noting that other mechanisms, such as repellency or direct intoxication of aphids by the essential oil treatment, could play a vital role in the reduction in virus transmission [42].

Active compounds that are present in essential oils and other plant extracts have different biological effects on phytophagous insects, including a broad spectrum of physiological dysfunctions. Determination of these changes in the insect body is an important method of identifying the toxicity mechanisms of the applied substances [43,44]. Insect proteases are essential digestive enzymes that catalyze the release of amino acids from dietary proteins. The impairment of its activity may lead to poor nutrient absorption and the non-availability of essential amino acids [45]. The present results showed that treatment with sublethal concentration of the tested EOs from Asteraceae plants lowered the activity of trypsin and pepsin in *R. padi* females. Similar supportive results were reported in diverse groups of insect pests after exposure to EOs and other plant extracts. Shahriari et al. [46] revealed that *Teucrium polium* essential oil decreased the activity of trypsin, chymotrypsin, and general proteases in *Ephestia kuehniella* (Z.). Active compounds from ginger (*Zingiber officinale*) extract inhibited the activity of pepsin, lipase, and α-amylase in sorghum aphid (*Melanaphis sorghi*) [47]. The authors also state that the inhibitory effect led to malnutrition of the insects and consequently limited their growth and development. Reduced enzyme activity may be due to biochemical inhibition by active compounds that are present in the extracts. It is well known that the inhibition by plant secondary metabolites such as phenolics results from their binding to nucleophilic sites of the enzymes [48]. Interestingly, our results indicated that the EOs with stronger deterrent activity and previously reported toxicity toward aphids [20] also evoked a higher level of protease inhibition. It suggests that enzyme inhibition may be a crucial factor contributing to the aphicidal activity of the tested EOs. On the other hand, the activity of proteases is strictly related to the feeding process, i.e., the amount of food that passes through the digestive tract. The imbalance in enzyme–substrate complex and inhibition of peristaltic movement of the gut can lead to a reduction in digestive enzyme activity [49]. Therefore, the lower activity of proteases in aphids after treatment with essential oils may be the result of feeding deterrence.

Digestive enzymes, such as α- and β-glucosidase play a significant role during terminal digestion of carbohydrates in the insect’s midgut. α-glucosidase is characterized by sucrase and transglucosidase activity and catalyzes both the production of monosaccharides and the synthesis of glucose-dominated oligosaccharides; thus, this enzyme plays a key role in carbon nutrition and osmoregulation in aphids [50]. β-glucosidase hydrolyses β-glycosidic linkages of oligosaccharides and glycosides to release non-reducing terminal glucosyl residues. Besides liberating monosaccharides that can be absorbed, this enzyme also affects insect–plant interaction, by hydrolysis of plant glycosides and release of toxic aglycones [51]. The analysis of α- and β-glucosidase activity revealed that treatment of the bird-cherry oat aphid with the tested EOs generally evoked a decrease in the enzyme activity, and the strongest effect was obtained after application of essential oils from *T. vulgare* and *S. chamaecyparissus*. Results analogous to the present findings were also reported by Magierowicz et al. [14] when they treated *A. advenella* larvae with *T. vulgare* essential oil and its main components. All the tested substances decreased α- and β-glucosidase activity within insect tissues, but the greatest decline was shown after treatment with thujone, which is the main component of tansy essential oil. Moreover, Khosravi and Sendi [52] reported that essential oils from garden thyme and lavender reduced the activity of these two glucosidases in the midgut of *Xanthogaleruca luteola* (Müller), and the observed effect was concentration-dependent. These authors also suggest that the toxicity mechanism of the applied preparations was associated with the disruption of carbon metabolism and/or osmoregulation in the insect body. It is generally believed that the most abundant constituents of essential oils determine their biological activity. Our results evidenced that despite significant differences in the chemical composition of the main constituents of the tested Asteraceae EOs, their effect on the activity of glucosidases and proteases in *R. padi* was quite similar. Such an effect could be explained by the multicomponent complexity of the EOs. The role of the remaining compounds in the mixture cannot be ignored because even the minor constituents may have a crucial function due to the coupled effects, additive action between metabolites as well as synergic or antagonistic interactions [53].

In summary, our analyses indicate that the studied EOs from Asteraceae plants possess anti-settling as well as feeding deterrent activity toward the bird cherry-oat aphid. Among them, the most active was the EO from *A. millefolium* followed by oils from *S. chamaecyparissus*, *T. vulgare* and *T. patula*. On treated plants, aphids started probing later and the probes were shorter. Additionally, almost all of the tested oils evoked a decrease in the total duration of phloem sap ingestion. The analyzed EOs, even at sublethal concentrations, also reduced the activity of proteases and glucosidases within aphid tissues. It suggests that the mechanism of action of the oils may be associated with their ability to inhibit the activity of key digestive enzymes in adult females of *R. padi*.

## 4. Materials and Methods

### 4.1. Aphid Culture

The bird cherry-oat aphid used in this study was obtained from a stock culture kept at the University of Siedlce, Poland. The stock culture was maintained on winter wheat seedlings (cv. Tonacja) in a controlled environmental chamber at 22 ± 1 °C with 65% relative humidity (RH) and L16:D8 photoperiod. Adult apterous females (2–3 days after the final molt) we used for all subsequent experiments.

### 4.2. Plant Material and EO Extraction

Essential oils were extracted from four plants of the Asteraceae family harvested at the flowering stage. The whole above-ground parts of plants (stems with leaves and flowers) were collected from native habitats (*A. millefolium*, *T. vulgare*) or from our own collection cultures (ornamental plants: *S. chamaecyparissus*, *T. patula*), (Siedlce district, 52°17′ N, 22°24′ E, Poland). The harvested material was dried in the shade at a temperature of 25–30 °C and then pulverized by manual grinding. In total, 50 g of the plant material along with 500 mL of water were subjected to hydro-distillation for 3 h using a Clevenger-type apparatus. The obtained oils were dried over anhydrous sodium sulfate, filtered and stored in amber vials at 4 °C until use.

### 4.3. Bioassays

All bioassays were carried out in controlled conditions at 22 ± 1 °C, 65% RH and L16:D8 photoperiod. The tested EOs were dissolved in ethanol to give stock solutions of 40% (*w*/*v*) concentration. The EO solutions for bioassays (0.2%, *w*/*v*) were prepared from the stock solution and distilled water with the addition of Tween 80 as an emulsifier (0.075% *v*/*v*) and 2% (*v*/*v*) ethanol. A mixture with the same composition, but lacking the EO, was used as a control solution.

#### 4.3.1. Settling Inhibition Bioassays

The settling inhibition potential of the studied Asteraceae EOs towards *R. padi* was assessed using a conventional settling choice test [54]. Leaves cut from wheat seedlings were dipped for 10 s in an essential oil solution (0.2%) or control solution and dried in the air for 30 min. at room temperature. Afterward, treated and control leaves were placed in Petri dishes (20 cm diameter) lined with moistened filter paper to avoid dryness (Figure S1). Twenty apterous females were placed in the center of the dish between the two leaves, and the number of aphids that settled on each leaf was recorded at 1, 4, 8 and 24 h intervals after access to the leaves. This experiment was replicated ten times for each treatment. A settling inhibition index (%SI) was calculated using the equation: %SI = [1 − (%T/%C)] × 100, where %T and %C are the percentage of aphids settled on the treated or control leaf, respectively [54].

#### 4.3.2. Electronic Registration of Aphid Probing Behavior

The probing behavior of the bird cherry-oat aphid on EO-treated and control plants was monitored using the Electrical Penetration Graphs (EPG) technique (Figure S2) [55]. This technique allows in situ assessment of the feeding behavior of piercing–sucking insects and is commonly applied in Hemiptera–plant relationship studies [38,56]. In the present study, adult apterous females of *R. padi* were attached to a golden wire electrode with conductive silver glue (EPG-Systems, Wageningen, The Netherlands) and starved for one hour prior to the experiment. Each aphid was given access to a freshly prepared plant: seven-day-old wheat seedlings were treated with the studied essential oil (0.2%, *w*/*v*) or control solution. The seedlings were dipped for 10 s in the appropriate mixture and dried in the air for 30 min at room temperature. The probing behavior of 16 aphids per treatment was monitored in a Faraday cage for 8 h. The following behavioral aphid activities were distinguished: np—non-probing, C—pathway phase, G—xylem phase, E—phloem phase (divided into E1 and E2 that represent watery salivation and sap ingestion, respectively) and pd—potential drops (short punctures of cells during C phase) [30]. The parameters derived from EPG recordings were analyzed according to their duration and frequency in configuration related to aphid probing activities in peripheral and vascular plant tissues. These parameters were measured in each of the test groups and recalculated per insect.

### 4.4. Insect Treatment and Biochemical Analyses

Three hundred apterous females were caged on 7 day old seedlings of winter wheat, and after 2 h, the aphids were sprayed with an essential oil solution at a concentration of 0.2% or with a control solution, prepared as described above. The solutions were applied with a laboratory sprayer at a rate of 10 mL per 30 seedlings settled by aphids. The applied sublethal concentration was selected based on our previous results concerning contact toxicity tests, where it caused, depending on the oil, between 20% and 35% mortality in aphids [19,20]. The effect of the tested essential oils on the activity of enzymes within *R. padi* tissues was assayed 12, 24, 48 and 72 h after exposure. Collected aphids were homogenized in ice-cold 0.1 M Tris-HCl buffer pH 7.0 for trypsin and pepsin assays or 0.2 M phosphate buffer pH 5.8 for α- and β-glucosidase. The homogenates were filtered through two layers of cheesecloth and centrifuged at 10,000× *g* for 20 min at 4 °C. The obtained supernatants were used in further analyses. All enzymatic assays were repeated independently at least three times.

#### 4.4.1. Trypsin Assay

Trypsin activity was assayed according to the method described by Pontual et al. [57], with slight modifications. The reaction mixture containing 0.1 mL of enzyme extract, 0.4 mL Tris-HCl buffer (pH 8.0) and 1.0 mL of BA*p*NA (Nα-benzoyl-DL-arginyl -*p*-nitroanilide, 0.1 mM) as substrate was incubated at 30 °C for 30 min. The enzymatic reaction was stopped by the addition of 0.5 mL 20% trichloroacetic acid (TCA). The absorbance was measured at 405 nm. The concentration of *p*-nitroaniline was calculated using an excitation coefficient of 9500 M^−1^ cm^−1^ and the enzyme activity was expressed as nmol of *p*-nitroaniline released × min^−1^ × mg^−1^ protein.

#### 4.4.2. Pepsin Assay

Pepsin activity was determined according to Anson [58] and Houseman and Downe [59], using 2% hemoglobin as substrate. For this purpose, 0.5 mL of the enzyme extract was incubated for 30 min at 30 °C with 1.0 mL glycine buffer (pH 2.0) and 0.5 mL hemoglobin. Proteolysis was stopped by the addition of 20% TCA, and after precipitation, the mixture was centrifuged for 15 min at 12,000× *g* under 4 °C. The amount of tyrosine liberated from hemoglobin was estimated using Folin–Ciocalteu reagent and expressed in nmol × min^−1^ × mg^−1^ protein.

#### 4.4.3. Assay of α- and β-Glucosidase

The activity of α- and β-glucosidases was determined using the Katagiri [60] method. The activities were measured with *p*-nitrophenyl-α-D-glucopyranoside (*p*NαG) or *p*-nitrophenyl-β-d-glucopyranoside (*p*NβG) as substrates for α- and β-glucosidase, respectively. The reaction mixture contained 0.2 mL aphid homogenates, 0.1 mL phosphate buffer pH 5.8 and 0.2 mL *p*NαG (15 mM solution in extraction buffer) or *p*NβG (50 mM solution in extraction buffer). The mixture was incubated for 60 min at 30 °C, and the reaction was stopped by the addition of 3 mL 2% sodium carbonate solution. The amount of released *p*-nitrophenol was measured spectrophotometrically at 405 nm. The activity of α- and β-glucosidases was expressed as nmol *p*-nitrophenol released × min^−1^ × mg^−1^ protein.

#### 4.4.4. Protein Content Measurement

The amount of protein within the aphid homogenates was determined after Bradford [61], using an acidic solution of Coomassie Brilliant Blue G-250 (Bio-Rad, Munich, Germany). The absorbance of the blue complex was measured at 595 nm; bovine serum albumin (BSA) was used as the standard.

### 4.5. Statistical Analysis

For data deriving from the leaf-choice bioassay, the Student’s *t*-test was used to analyze the differences between the number of *R. padi* on treated leaves and the number of insects on control leaves. The parameters of aphid probing behavior on EO-treated plants were compared pair-wise with the control plant by a non-parametric Mann–Whitney U test. Enzyme activity was analyzed using repeated measures ANOVA with EO and time as fixed effects. The significance of differences between mean values was calculated by Tukey’s multiple comparisons test at *p* < 0.05. All statistical analyses were carried out using Statistica v. 13.3 software (Statsoft, Krakow, Poland).

## Figures and Tables

**Table 1:** Effect of essential oils on the settling behavior of *Rhopalosiphum padi*.

Essential Oil	Time (h)	Number of Aphids per Leaf		*p*	% SI
		Treated	Control		
*A. millefolium*	1	2.2 ± 0.46	11.6 ± 0.94	<0.001	81.0
	4	3.1 ± 0.90	13.9 ± 0.92	<0.001	77.7
	8	4.1 ± 0.60	14.1 ± 0.92	<0.001	70.9
	24	4.3 ± 0.77	13.6 ± 0.91	<0.001	68.4
*S. chamaecyparissus*	1	2.5 ± 0.68	10.2 ± 1.05	<0.001	75.5
	4	3.8 ± 1.01	12.6 ± 1.11	<0.001	69.8
	8	4.2 ± 0.97	13.3 ± 1.25	<0.001	68.4
	24	4.8 ± 1.17	13.3 ± 1.32	<0.001	63.9
*T. patula*	1	5.4 ± 1.02	10.4 ± 1.21	0.005	48.1
	4	5.2 ± 0.94	12.2 ± 0.95	<0.001	57.4
	8	6.4 ± 0.68	12.6 ± 0.94	<0.001	49.2
	24	7.4 ± 0.82	10.9 ± 0.84	0.008	32.1
*T. vulgare*	1	2.2 ± 0.29	6.5 ± 0.99	<0.001	66.1
	4	4.8 ± 0.64	11.4 ± 0.67	<0.001	57.9
	8	4.6 ± 0.65	11.4 ± 0.73	<0.001	59.6
	24	5.2 ± 0.80	11.8 ± 1.04	<0.001	55.9

Numbers represent the mean number (±SE) of aphids that settled on the treated and control leaves; *p* < 0.05 denotes statistically significant differences (Student *t*-test); % SI—settling inhibition activity in percentage.

**Table 2:** Probing behavior of *Rhopalosiphum padi* on wheat seedlings treated with selected Asteraceae essential oils.

EPG Parameter	Control	Essential Oils			
		*A. millefolium*	*S. chamaecyparissus*	*T. patula*	*T. vulgare*
General Aspects of Aphid Probing Behavior					
Time from start of EPG to first probe (min)	6.3 ± 1.4	23.9 ± 6.3 *	15.7 ± 3.1 *	10.6 ± 2.5	8.0 ± 1.2
Number of probes	6.9 ± 0.8	11.1 ± 1.7 *	10.1 ± 1.8	7.9 ± 1.4	14.4 ± 2.6 *
Total duration of probing (min)	451.8 ± 4.6	404.2 ± 12.0 *	426.2 ±7.8 *	434.0 ± 6.0 *	441.3 ± 6.3
Number of pathway phases	10.4 ± 1.0	15.3 ± 1.6 *	14.5 ± 1.7 *	11.2 ± 1.5	18.8 ± 2.5 *
Total duration of pathway phase (min)	121.2 ± 12.8	173.5 ± 21.4	152.6 ± 20.3	145.7 ± 19.9	216.4 ± 23.6 *
Total duration of xylem phase (min)	7.8 ± 3.9	11.9 ± 4.2	5.3 ± 3.0	9.8 ± 4.5	14.8 ± 5.1
Total duration of phloem phase ^a^ (min)	322.8 ± 17.8	218.7 ± 29.7 *	268.2 ± 19.0	278.4 ± 19.7	210.1 ± 26.7 *
Phloem phase index ^b^	0.71 ± 0.03	0.48 ± 0.06 *	0.67 ± 0.05	0.65 ± 0.05	0.49 ± 0.07 *
Aphid Probing Behavior Associated with Phloem Phase					
Total duration of phloem salivation phase (min)	12.2 ± 1.9	13.8 ± 2.5	23.0 ± 2.0 *	13.4 ± 1.6	17.1 ± 2.0
Time from start to first salivation ^c^ (min)	65.6 ± 11.6	94.8 ± 14.4	103.8 ± 18.9	79.8 ± 10.9	142.2 ± 16.6 *
Total duration of phloem ingestion (min)	310.6 ± 17.8	205.0 ± 28.9 *	245.2 ± 19.5 *	265.0 ± 19.5	193.0 ± 26.7 *
Time from start to first sustained phloem ingestion ^d^ (min)	104.4 ± 14.7	151.4 ± 19.9	136.1 ± 20.6	118.8 ± 12.9	184.3 ± 18.2 *
Number of probes before the first sustained phloem ingestion ^d^	4.7 ± 0.6	6.1 ± 0.7	5.4 ± 0.7	5.2 ± 0.8	7.1 ± 1.3
Potential drops (pd)					
Total number of pd	66.3 ± 6.6	79.8 ± 7.7	70.3 ± 8.9	68.1 ± 8.6	88.1 ± 7.2 *
Mean duration of a single pd (s)	4.3 ± 0.07	4.1 ± 0.12	4.5 ± 0.13	4.4 ± 0.08	4.3 ± 0.09
Total duration of sub-phase pd II-1 (min)	1.9 ± 0.24	2.0 ± 0.20	2.0 ± 0.27	1.9 ± 0.24	2.3 ± 0.22
Total duration of sub-phase pd II-2 (min)	1.1 ± 0.14	1.4 ± 0.14	1.3 ± 0.18	1.0 ± 0.16	1.5 ± 0.15
Total duration of sub-phase pd II-3 (min)	1.7 ± 0.20	2.2 ± 0.23	1.9 ± 0.27	1.7 ± 0.23	2.4 ± 0.22 *

Values are means ± SE; means followed by asterisk indicate a significant difference in relation to control (*p* < 0.05, Mann–Whitney U test); ^a^ (E1 + E2); ^b^ (E1 + E2)/(C + E + G); ^c^ only aphids that showed at least one phloem phase E1 were included in analysis; ^d^ only aphids that showed at least one phloem phase E2 > 10 min were included in analysis.

## Data Availability

The data presented in this study are available in the article.

## Supplementary Materials

The following supporting information can be downloaded at: https://www.mdpi.com/article/10.3390/molecules29071673/s1, Figure S1: Bioassays of settling inhibition in *Rhopalosiphum padi*; Figure S2: Analysis of aphid feeding behavior by electronic penetration graph (EPG) technique.
