# Supplementary material for: The Effect of Essential Oils from Asteraceae Plants on Behavior and Selected Physiological Parameters of the Bird Cherry-Oat Aphid
Source: Molecules. 2024 Apr 8;29(7):1673. doi: 10.3390/molecules29071673 (PMC11013816; doi:10.3390/molecules29071673)
Supplement: Supplementary file 1 [file molecules-29-01673-s001.zip › molecules-2914813-supplementary.pdf]

Supplementary Material

The Effect of Essential Oils from Asteraceae Plants on Behavior and Selected Physiological Parameters of the Bird Cherry-Oat Aphid

Paweł Czerniewicz [1,*], Hubert Sytykiewicz [1] and Grzegorz Chrzanowski [2]

[1] Institute of Biological Sciences, Faculty of Natural Sciences, University of Siedlce, Prusa 14, 08-110 Siedlce, Poland; hubert.sytykiewicz@uws.edu.pl

[2] Institute of Biotechnology, University of Rzeszow, Zelwerowicza 8B, 35-601 Rzeszow, Poland; gchrzanowski@ur.edu.pl

* Correspondence: pawel.czerniewicz@uws.edu.pl; Tel.: +48-25-643-1389

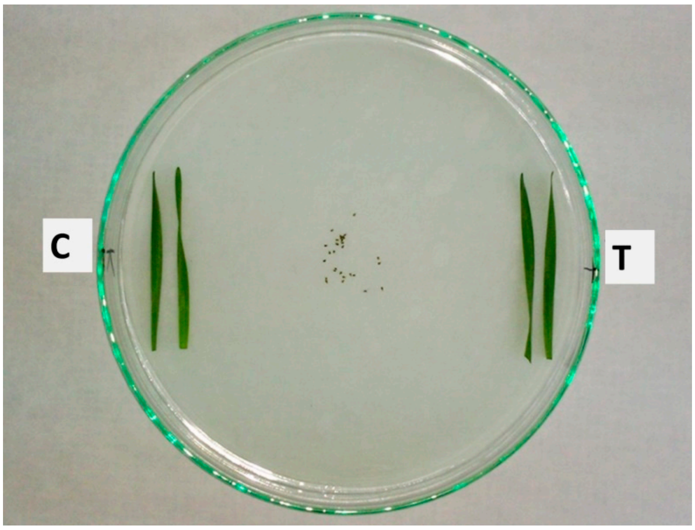

Figure S1. Bioassays of settling inhibition in *Rhopalosiphum padi*; C—control leaves; T—treated leaves; aphid females were placed in the centre of Petri dish (20 cm diameter).

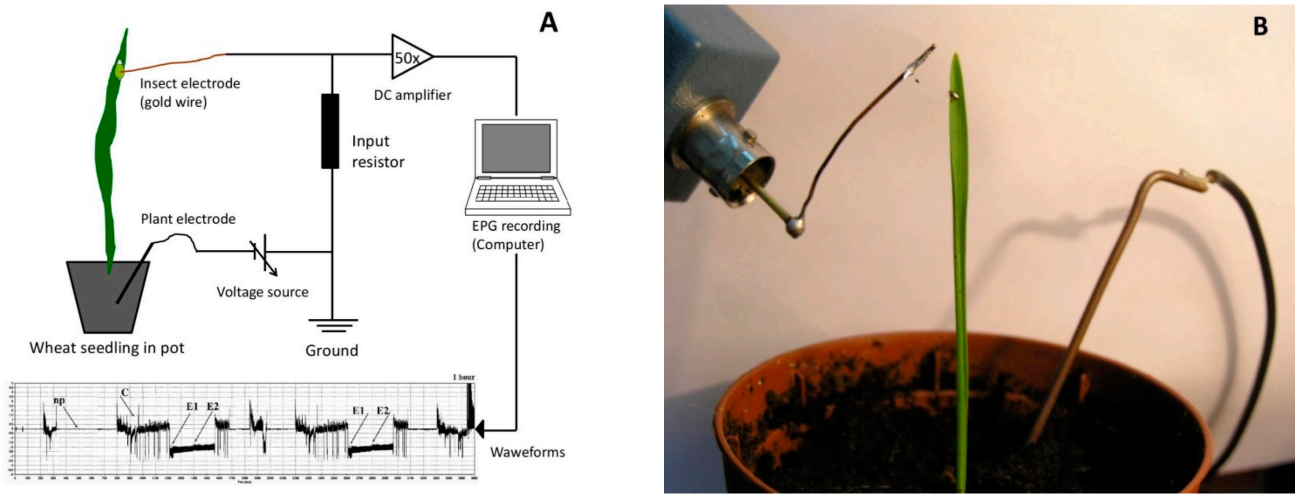

Figure S2. Analysis of aphid feeding behaviour by electronic penetration graph (EPG) technique; (A) EPG recording schematic diagram; (B) Bird cherry-oat aphid on wheat seedling during EPG recording.
